# Non-native fish of the Upper Irtysh and the Ulungur Rivers in China

**DOI:** 10.3897/BDJ.11.e97884

**Published:** 2023-01-17

**Authors:** Chen Tian, Lei Fang, Xuejian Li, Yonghua Li, Tianjian Song, Jiang Chang, Cunqi Liu, Yahui Zhao

**Affiliations:** 1 College of life sciences, Hebei University, Baoding, China College of life sciences, Hebei University Baoding China; 2 Key Laboratory of the Zoological Systematics and Evolution, Institute of Zoology, Chinese Academy of Sciences, Beijing, China Key Laboratory of the Zoological Systematics and Evolution, Institute of Zoology, Chinese Academy of Sciences Beijing China; 3 State Key Laboratory of Environmental Criteria and Risk Assessment, Chinese Research Academy of Environmental Sciences, Beijing, China State Key Laboratory of Environmental Criteria and Risk Assessment, Chinese Research Academy of Environmental Sciences Beijing China; 4 Institute of Shandong River Wetlands, Jinan, China Institute of Shandong River Wetlands Jinan China

**Keywords:** Altay region, distribution, non-native fish, endangered species

## Abstract

**Background:**

The Chinese section of the Irtysh River Basin and the Ulungur River Basin, two major river basins of the Altay region, are located at the northwest of Xinjiang Uygur Autonomous Region of China. As an international river, the Chinese section has seven state-level protected fish and seven local-level protected species as well. Many more commercial species have been introduced from eastern China and other countries, accompanied by some low-value and small-sized fish in recent decades. The non-native fish species have already threatened these protected fish. This study investigated the distribution of non-native fish species in the Chinese section of the Irtysh River Basin and the Ulungur River Basin. The basic data for the biodiversity conservation and the information of the non-native fish in these two river basins were gathered.

**New information:**

There are a lot of studies on native fish in the Chinese section of the Irtysh River Basin and Urungur River Basin in China, but there is a lack of studies on non-native fish. Thirteen non-native fish belonging to four orders, nine families and 12 genera were collected in this study. The study includes one dataset. The dataset presents taxonomy, distribution, water body and location for each of the non-native fish collected from the Chinese section of the Irtysh River Basin and the Ulungur River Basin. Our study has found that the proportion of native species has declined, while the number of non-native species has increased from 2013 to 2022. The information we provided could help to develop an international strategy for the protection of aquatic biodiversity.

## Introduction

The Altay region, bordering Russia, Kazakhstan and Mongolia, is located in the northern part of the Xinjiang Uygur Autonomous Region of China. The Irtysh River and the Ulungur River (http://www.feow.org/ecoregions/details/603) are two important rivers in the Altay region. The Irtysh River, an essential international river, is the second largest river in Xinjiang and it is also the only river in China that flows into the Arctic Ocean (Duman and Nadila 2017). The Irtysh River originates from the southern slope of the Altai Mountains and the confluence of the Kayierte River and the Kuyierte River forms the Irtysh River in Koktokay (Dong and Li 2010). The total length of the Irtysh River is 4,248 km, of which the length of the Chinese section is 546 km, with a drainage area of 57,000 km^2^ and an annual runoff of approximately 11.1 billion m^3^ (Li et al. 2020). The main tributaries of the Chinese section of the Irtysh River that includes the Kelan, Buerjin, Haba and Bieliezeke Rivers, join from the north bank, making the Irtysh River Basin as a "comb-shaped" water system (Ren et al. 2002).

The Ulungur River, the second largest river in the Altay region, originates from the Altai Mountains in Qinghe County, with a total length of 821 km, a drainage area of 61,400 km^2^ and an annual runoff of about 1.07 billion m^3^ (Nurlan 2014). The Irtysh River and the Ulungur River were originally two independent water systems in Late Pleistocene (Wang 2010). Due to the construction of a 3 km-long canal in 1986-1987, the Ulungur Lake, into which the Ulungur River drains, has become a subsidiary water body of the Irtysh River (Liu 2015).

A historical survey reported 22 native fish species in the upper reaches of the Irtysh River (Chinese section) and the Ulungur River Basins (Li et al. 1966, Guo et al. 2003, Huo et al. 2010, Yang et al. 2016). Although they only constitute 1.6% amongst the 1363 native fishes in China, they are very essential and unique in the distribution of inland fish in China (Zhang and Zhao 2016). Seven fish species have been listed as nationally protected species, i.e. *Acipenserbaerii*, *Acipenserruthenus*, *Huchotaimen*, *Brachymystaxlenok*, *Stenodusleucichthys*, *Thymallusarcticus* and *Lethenteroncamtschaticum*. Non-native fish species have been suggested as being one of the most important factors that threaten biodiversity (Vitule et al. 2009, Reid et al. 2019, Su et al. 2021). To further protect native fish species in this area, it is imperative to monitor the distribution and current status of non-native fish species. Therefore, we conducted surveys on non-native fish species of the Chinese section of the Irtysh River Basin and the Ulungur River Basin from 2013 to 2022. The results provide basic data for the comprehensive assessment and protection of native fish diversity in the basins.

## Sampling methods

### Sampling description

**Figure 1.**
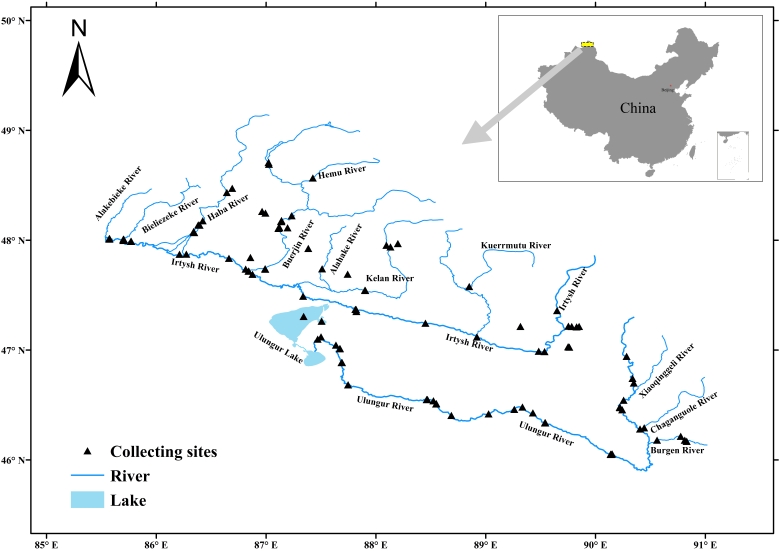
Location of the sampling sites.

Fish specimens were collected at 46 sites of the Irtysh River Basin and 30 sites of the Ulungur River Basin from 2013 to 2022 (Fig. 1). Samples were collected by hand net (dense mesh), cast net (aperture 1 cm×1 cm, diameter 5 m, length 3.5 m) and traps (aperture 0.5 cm×0.5 cm, length 10 m) in a variety of aquatic environments. Samples were collected by hand net and cast net in fast-flowing and shallow waters. Traps were used in slow-flowing waters and pools. Four traps were usually placed at the sampling point for 12 h overnight. The collection time using the hand nets is one hour. When using cast-net sampling, a single sampling point is collected for 30 minutes. The specimens were fixed in 95% ethanol or 10% formaldehyde solution in the field. Coordinates of sampling localities were recorded by hand-held GPS locator. The specimens were stored in 75% ethanol in the National Zoological Museum, Institute of Zoology, Chinese Academy of Sciences (ASIZB).

### Step description

Fish species were identified by two professional fish taxonomists each time and referred to literature on fish species of the Irtysh River and the Ulungur River (Institute of Zoology of Chinese Academy of Sciences et al. 1979, Wu 1982, Chen 1998, Le 2000, Wu and Zhong 2008). Valid species names were in accordance with the taxonomic literature (Zhang et al. 2020, Fricke et al. 2022).

## Geographic coverage

### Description

We surveyed the Chinese section of the Irtysh River Basin and the Ulungur River Basin, covering various habitats, including swift-flowing waters, running waters and pools. The collection sites were marked by ArcGIS 10.2 software.

### Coordinates

46.050 and 48.2233627 Latitude; 90.83 and 85.5708 Longitude.

## Taxonomic coverage

### Description

**Figure 2.**
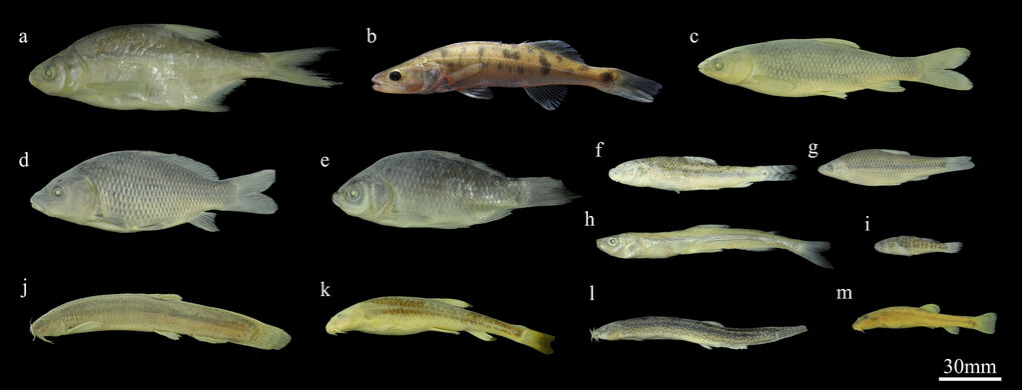
Specimen photos of some fish species collected from the Irtysh River Basin (Chinese section) and the Ulungur River Basin. **a** *Abramisbrama* (Linnaeus, 1758); **b** *Sanderlucioperca* (Linnaeus, 1758); **c** *Ctenopharyngodonidella* (Valenciennes, 1844); **d** *Cyprinuscarpio* Linnaeus, 1758; **e** *Carassiusauratus* (Linnaeus, 1758); **f** *Abbottinarivularis* (Basilewsky, 1855); **g** *Pseudorasboraparva* (Temminck & Schlegel, 1846); **h** *Hypomesusolidus* (Pallas, 1814); **i** *Micropercopsswinhonis* (Günther, 1873); **j** *Misgurnusdabryanus* (Dabry de Thiersant, 1872); **k** *Triplophysastrauchii* (Kessler, 1874); **l** *Misgurnusanguillicaudatus* (Cantor, 1842); **m** *Hedinichthysminuta* (Li, 1966).

In total, four orders, nine families, 12 genera and 13 non-native fish were collected in the Irtysh River Basin (Chinese section) and the Ulungur River Basin. Specimen photos of the non-native fish species are presented in Fig. 2.

### Taxa included

**Table:** 

Rank	Scientific Name	
kingdom	Animalia	
phylum	Chordata	
class	Actinopterygii	
order	Cypriniformes	
order	Perciformes	
order	Gobiiformes	
order	Osmeriformes	
family	Cobitidae	
family	Cyprinidae	
family	Gobionidae	
family	Leuciscidae	
family	Nemacheilidae	
family	Percidae	
family	Odontobutidae	
family	Osmeridae	
family	Xenocyprididae	
subfamily	Cyprininae	
subfamily	Leuciscinae	
subfamily	Luciopercinae	
genus	*Abbottina* Jordan et Fowler, 1903	
genus	*Abramis* Cuvier, 1816	
genus	*Ctenopharyngodon* Steindachner, 1866	
genus	*Cyprinus* Linnaeus, 1758	
genus	*Carassius* Jarocki, 1882	
genus	*Hypomesus* Gill, 1862	
genus	*Micropercops* Fowler et Bean, 1920	
genus	*Misgurnus* Lacépède, 1803	
genus	*Pseudorasbora* Bleeker, 1860	
genus	*Sander* Oken, 1817	
genus	*Triplophysa* Rendahl, 1933	
genus	*Hedinichthys* Rendahl, 1933	
species	*Abbottinarivularis* (Basilewsky, 1855)	
species	*Abramisbrama* (Linnaeus, 1758)	
species	*Ctenopharyngodonidella* (Valenciennes, 1844)	
species	*Cyprinuscarpio* Linnaeus, 1758	
species	*Carassiusauratus* (Linnaeus, 1758)	
species	*Hypomesusolidus* (Pallas, 1814)	
species	*Micropercopsswinhonis* (Günther, 1873)	
species	*Misgurnusanguillicaudatus* (Cantor, 1842)	
species	*Misgurnusdabryanus* (Dabry de Thiersant, 1872)	
species	*Pseudorasboraparva* (Temminck et Schlegel, 1846)	
species	*Sanderlucioperca* (Linnaeus, 1758)	
species	*Triplophysastrauchii* (Kessler, 1874)	
species	*Hedinichthysminuta* (Li, 1966)	

## Temporal coverage

**Data range:** 2013-9-04 – 2013-9-11; 2014-9-12 – 2014-9-18; 2015-7-19 – 2015-7-24; 2016-7-16 – 2016-7-24; 2022-7-01 – 2022-7-24.

## Usage licence

### Usage licence

Creative Commons Public Domain Waiver (CC-Zero)

## Data resources

### Data package title

Non-native fish of the Upper Irtysh and the Ulungur Rivers in China

### Number of data sets

1

### Data set 1.

#### Data set name

Non-native fish of the Upper Irtysh and the Ulungur Rivers in China

#### Data format

Darwin Core

#### Download URL

https://ipt.pensoft.net/archive.do?r=x-j&v=1.0

#### Description

The dataset presents 13 non-native fish detected in the Chinese section of the Irtysh River Basin and the Ulungur River Basin, with a total of 151 data records and the number of fish being 2417. The important information including taxonomic, geographic location of the occurrence, water body and event date were provided for 13 non-native fish species (Suppl. material 1).

**Data set 1.** 

Column label	Column description
occurrenceID	An identifier for the Occurrence.
catalogNumber	An identifier for collected specimens.
basisOfRecord	The specific nature of the data record.
eventDate	The date during which an Event occurred.
scientificName	The full scientific name.
kingdom	The full scientific name of the kingdom in which the taxon is classified.
phylum	The full scientific name of the phylum in which the taxon is classified.
class	The full scientific name of the class in which the taxon is classified.
order	The full scientific name of the order in which the taxon is classified.
family	The full scientific name of the family in which the taxon is classified.
subfamily	The full scientific name of the subfamily in which the taxon is classified. No subfamily is represented by NA.
genus	The full scientific name of the genus in which the taxon is classified.
taxonRank	The taxonomic rank of the most specific name in the scientific Name as it appears in the original record.
ownerInstitutionCode	The name (or acronym) in use by the institution having ownership of the object(s) or information referred to in the record.
individualCount	The number of individuals represented present at the time and location of the Occurrence.
recordedBy	A list (concatenated and separated) of names of people, groups or organisations who record the information of the Taxon when collected.
identifiedBy	A list (concatenated and separated) of names of people, groups or organisations who assigned the Taxon to the subject.
decimalLatitude	The geographic latitude (in decimal degrees, using the spatial reference system given in geodeticDatum) of the geographic centre of a Location.
decimalLongitude	The geographic longitude (in decimal degrees, using the spatial reference system given in geodeticDatum) of the geographic centre of a Location.
maximumElevationInMetres	The geographic elevation (in metres, using the spatial reference system given in geodeticDatum) of the geographic centre of a Location.
geodeticDatum	The geographic information system (GIS) upon which the geographic coordinates given in decimalLatitude, decimalLongitude and metreElevation are based.
coordinateUncertaintyInMetres	The horizontal distance (in metres) from the given decimalLatitude and decimalLongitude describing the smallest circle containing the whole of the Location. Leave the value empty if the uncertainty is unknown, cannot be estimated or is not applicable (because there are no coordinates). Zero is not a valid value for this term.
locality	The specific description of the county from where specimens are collected.
country	The name of the country or major administrative unit in which the Location occurs.
stateProvince	The name of the next smallest administrative region than country (state, province, canton, department, region etc.) in which the Location occurs.
municipality	The full, unabbreviated name of the next smallest administrative region than county (city, municipality etc.) in which the Location occurs.
waterBody	The name of the water body in which the Location occurs.

## Additional information

**Figure 3.**
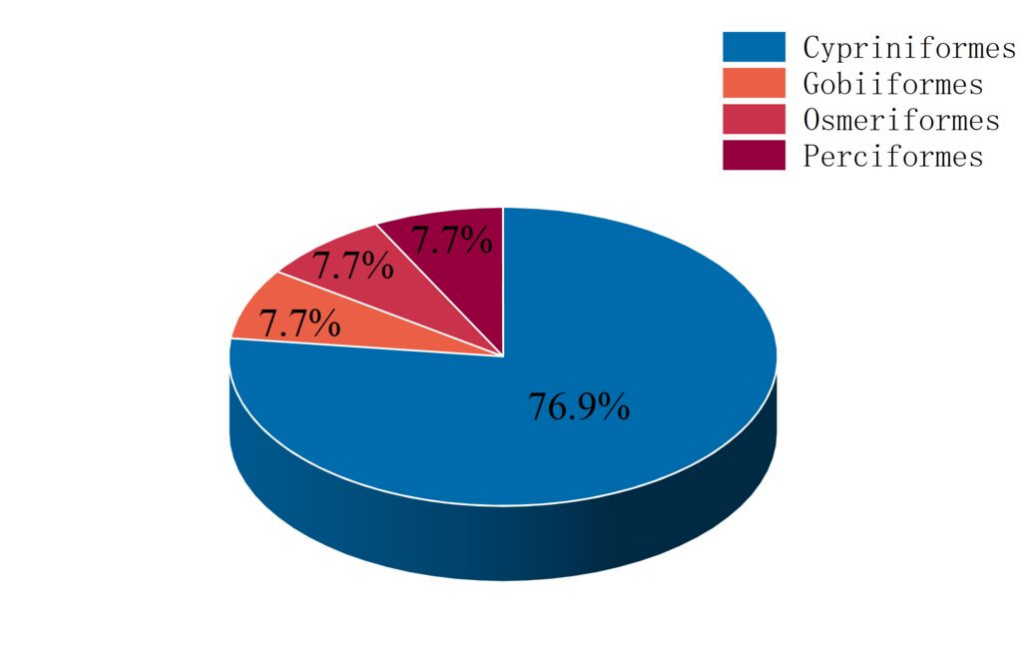
The fish composition of the Chinese section of the Irtysh River Basin and the Ulungur River Basin at the order level.

Thirteen non-native fish species in these two river basins account for 44.8% of the total fish species that we surveyed. Cypriniformes is the predominant order, accounting for 76.9% of the total number of non-native fish species. Perciformes, Gobiiformes and Osmeriformes account for 7.7% of the total number of non-native fish species, respectively (Fig. 3).

The introduction information about the 13 non-native fish species in this survey is shown in Table 1. *Cyprinuscarpio* was introduced to Lake Zaysan of the Irtysh River by the former Soviet Union in 1934 and 1935 and then it spread to the Irtysh River of China (Guo et al. 2003). *Abramisbrama* and *Sanderlucioperca*, both naturally distributed in the Caspian Sea Basin (Fricke et al. 2022), were introduced to the Iset River Basin of Russia by the former Soviet Union from 1959 to 1964 (Ren et al. 2002). Then they rapidly expanded to the Chinese section of the Irtysh River and Ulungur Lake after its artificial connection to the Irtysh River in 1970 (Ren et al. 2002). *Hypomesusolidus* is naturally distributed in the Arctic Ocean and North Pacific (Fricke et al. 2022). In April 1989, *Hypomesusolidus* of Shuifeng Reservoir in Liaoning Province was successfully transplanted in Chaiwopu Lake in Urumqi, Xinjiang and then it was transplanted to the Ulungur Lake and other waters of Xinjiang in 1991 (Ren et al. 2002, Chen 2012). In 2013 and 2014, we recorded *Misgurnusdabryanus* and *Misgurnusanguillicaudatus* in the Irtysh River Basin for the first time, but not in the Ulungur River Basin. In the 2022 survey, *Misgurnusdabryanus* and *Misgurnusanguillicaudatus* were already recorded in the Ulungur River Basin. *CtenopharyngodonIdella* and *Carassiusauratus* have been transplanted to the Irtysh River and Ulungur Lake since the 1980s. Meantime, some small-sized fish, such as *Pseudorasboraparva*, *Abbottinarivularis* and *Micropercopsswinhonis*, were also brought with those commercial species from eastern China (Jiang and Huo 2013). *Triplophysastrauchii* and *Hedinichthysminuta* that found their way into the Irtysh River Basin may have been caused by the introduction of *Cyprinuscarpio* and *Carassiusauratus* from Turpan Fishery in Xinjiang Uygur Autonomous Region in the 1990s (Xie et al. 2021). To sum up, three fish species were introduced from abroad amongst the 13 non-native fish in the Chinese section of the Irtysh River and Ulungur River Basins, including *Cyprinuscarpio*, *Abramisbrama* and *Sanderlucioperca*. Ten fish species were introduced from other rivers of China, including *Misgurnusdabryanus*, *Misgurnusanguillicaudatus*, *CtenopharyngodonIdella*, *Carassiusauratus*, *Hypomesusolidus*, *Pseudorasboraparva*, *Abbottinarivularis* and *Micropercopsswinhonis*, *Triplophysastrauchii* and *Hedinichthysminuta*, with the first five being active introduction and the last five being passive introduction.

**Figure 4.**
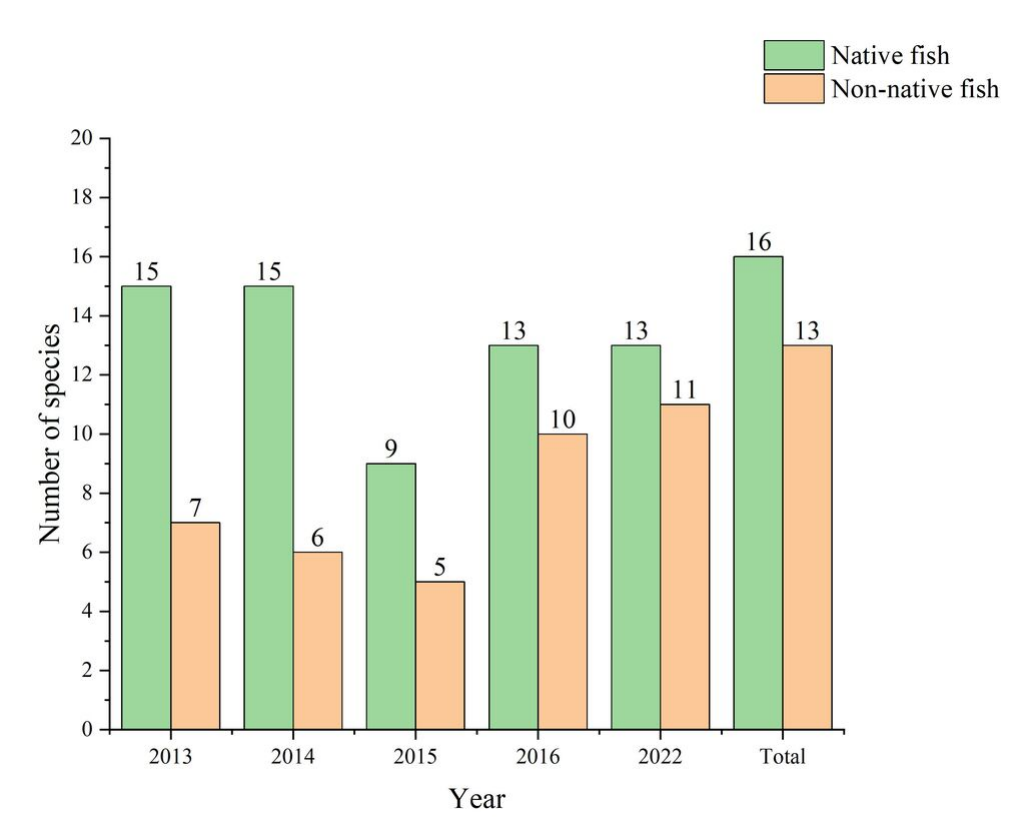
The number of native fish species and non-native fish species in different years in the the Chinese section of the Irtysh River Basin and the Ulungur River Basin.

**Figure 5.**
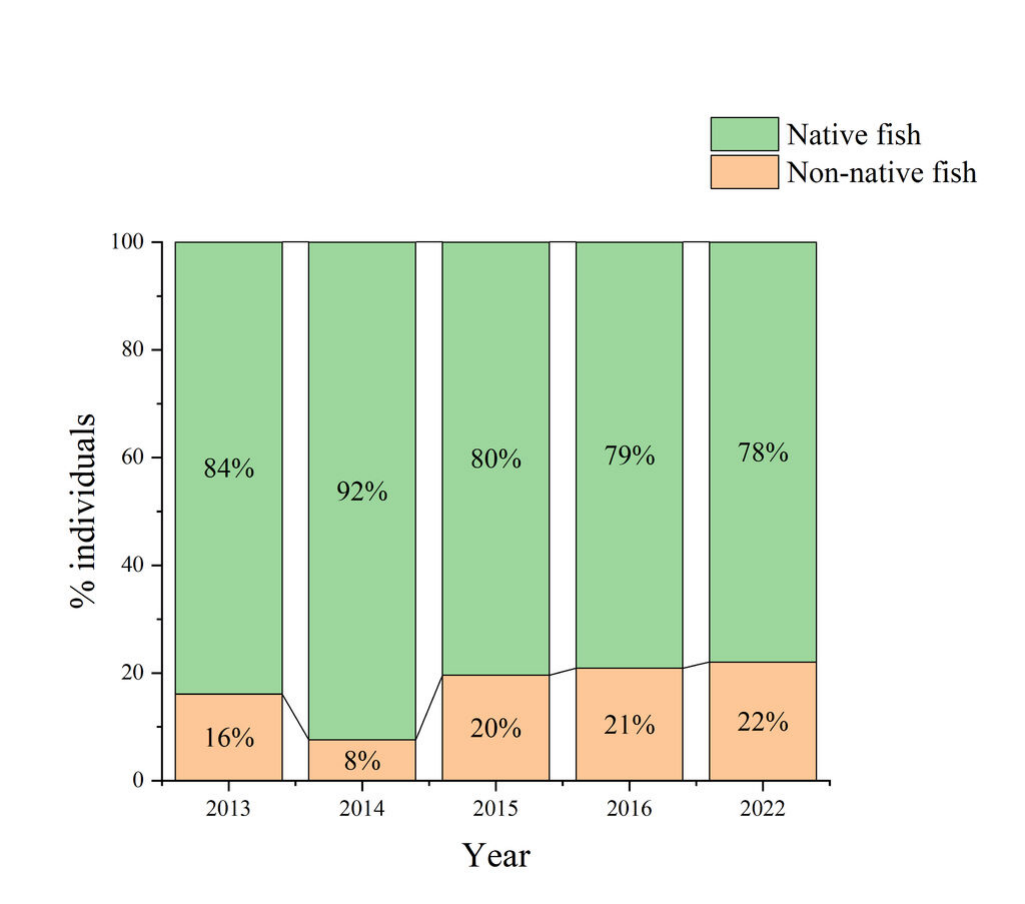
The proportion of native fish quantity and non-native fish to total quantity in different years in the the Chinese section of the Irtysh River Basin and the Ulungur River Basin.

Niche overlap is usually high between native species and non-native species and intense competition may cause the rapid decline and even the extinction of native populations (Hardin 1960, Bøhn et al. 2008). The highly invasive *Pseudorasboraparva* and *Carassiusauratus* encroach on the ecological niche of native fish to some extent (Zhang et al. 2014). The natural hybrids of the *Abramisbrama* and *Rutiluslacustris* are all over the Irtysh River and Ulungu Lake (Jiang and Huo 2013). The genetic exchange between the *Abramisbrama* and *R.lacustris* may lead to the genetic erosion or even the extinction of the *R.lacustris* (Zeng 2004, Xu et al. 2004). The population of *Percafluviatilis* has declined sharply due to the increasing number of *Hypomesusolidus* that feed on fish eggs in the Ulungur Lake (Tang et al. 2009, Chen 2012). The length range of *Leuciscusbaicalensis* was 19-23 cm and the weight range was 100-180 g in 1961, while the length range of this fish had decreased to 10-15 cm and the average weight range was only 100 g by the 1980s (Wang 1984). This is not only due to the overfishing, but also due to the introduction of non-native species. A total of 16 native fish and 13 non-native fish were investigated in the Chinese section of the Irtysh River and the Ulungur River Basins from 2013 to 2022. Compared with the historical records (22 native species), the number of native species decreased, while non-native species increased (Fig. 4). The number of individuals of non-native fish accounted for an increasing proportion of total catches during the survey period (Fig. 5). The decline in the number of native species is partly due to an increase in non-native species. There are seven rare and endangered fish species in the Chinese section of the Irtysh River Basin and the Ulungur River Basin, i.e. *Acipenserbaerii*, *Acipenserruthenus*, *Huchotaimen*, *Stenodusleucichthys*, *Thymallusarcticus*, *Cottusdzungaricus* and *Brachymystaxlenok* (Zhang and Cao 2021, IUCN 2022). These endangered species account for 31.8% of the total number of native fish species (22 species) in Altay region. There are 14 national and local protected species in total, accounting for 63.6% of the native species in Altay region (Table 2). The proportion of the endangered fish species in the two Basins are even higher than some major rivers basins in China, such as the Yangtze and Yellow Rivers (Cao et al. 2016), indicating that the protection of fish in the Altay region is important in Xinjiang and even to China as a whole. In the future, with the increasing species and quantity of non-native species and the construction of some water conservancy projects, native species will face great pressure to survive. The data we provided are helpful to understand the current distribution of non-native species in Altay region. It can complement the database of non-native species of Xinjiang and even in the country, which plays an essential role in dealing with non-native species invasion and ecological protection construction. Therefore, it is essential to continuously monitor these non-native fish in the Upper Irtysh and the Ulungur Rivers in China and to further explore the specific impacts of these non-native species.

## Supplementary Material

A30B0A54-E795-5F49-BDAA-3C96CEAE4EF1 10.3897/BDJ.11.e97884.suppl1 Supplementary material 1 Fish taxon-occurrences Data type taxon-occurrences Brief description Collected non-native fish taxon-occurrences of the Chinese section of Irtysh River Basin and Ulungur River Basin, Xinjiang, China. File: oo_773047.csv https://binary.pensoft.net/file/773047 authors of this paper

## Figures and Tables

**Table 1.** The introduction information about the 13 non-native fish species investigated.

Species	Introduction place		Introduction way		Introduction time
	Domestic	Foreign	Active	Passive	
*Cyprinuscarpio*		+	+		1934-1935
*Abramisbrama*		+	+		1959-1970
*Sanderlucioperca*		+	+		1959-1970
*CtenopharyngodonIdella*	+		+		1980s
*Carassiusauratus*	+		+		1980s
*Hypomesusolidus*	+		+		1991
*Pseudorasboraparva*	+			+	1980s
*Abbottinarivularis*	+			+	1980s
*Micropercopsswinhonis*	+			+	1980s
*Triplophysastrauchii*	+			+	1990s
*Hedinichthysminuta*	+			+	1990s
*Misgurnusdabryanus*	+		+		Uknown
*Misgurnusanguillicaudatus*	+		+		Uknown

**Table 2.** The information about 14 protected species in the Altay region.

Species	Protection level		Red List of China’s Vertebrates level
	National	Local	
Acipenserbaerii*	+		CR
Acipenserruthenus*	+		EN
*Huchotaimen*	+		EN
*Brachymystaxlenok*	+		EN
Stenodusleucichthys*	+		RE
Thymallusarcticus*	+		VU
Tincatinca*		+	LC
Leuciscusidus*		+	LC
Gymnocephaluscernua*		+	
Cottusdzungaricus*		+	VU
Rutiluslacustris*		+	LC
*Lethenteroncamtschaticum*	+		LC
CarassiusCarassius*		+	LC
*Lotalota*		+	LC
* In China, it is only found naturally in the Chinese section of the Irtysh River and the Ulungur River Basins; RE: Regionally Extinct, CR: Critically Endangered, EN: Endangered, VU: Vulnerable, LC: Least Concern, DD: Data Deficient.			
